# Genetic Assignment at Different Geographical Levels: A Case Study in a Forest Tree Species (*Pinus pinaster* Ait.) Using SNP Markers

**DOI:** 10.1111/eva.70145

**Published:** 2025-12-02

**Authors:** Sanna Olsson, Delphine Grivet, Marjana Westergren, Santiago C. González‐Martínez, Ricardo Alía, Juan José Robledo‐Arnuncio

**Affiliations:** ^1^ Instituto de Ciencias Forestales (ICIFOR‐INIA) Consejo Superior de Investigaciones Cientificas (CSIC) Madrid Spain; ^2^ Slovenian Forestry Institute Ljubljana Slovenia; ^3^ INRAE, Univ. Bordeaux, BIOGECO Cestas France

**Keywords:** gene pool, genetic assignment, maritime pine, origin identification, region of provenance, SNP markers

## Abstract

Genetic markers can assist in the identification of the stock origin in different organisms. Comparative studies of forest tree provenances have demonstrated that forest tree populations differ in performance across environments and at multiple geographic levels: populations nested within regions nested within gene pools. These levels are critical for conservation and sustainable use of genetic resources: regions of provenance are key units for seed marketing, while populations guide reproductive material collection under most seed regulations. Despite their potential, genetic methods have rarely been applied to identify forest tree origins due to methodological (sufficient number of highly discriminatory markers) and practical (construction of a baseline composed of a representative selection of samples) challenges. In our study, we analyzed a genomic dataset comprising 10,185 SNPs from 1579 samples of *Pinus pinaster* , a species with strong population structure, across 86 populations, 45 regions of provenance, and 10 gene pools, to discriminate among these hierarchical levels and assign individuals to them. We used two software packages to evaluate the reliability of our baseline dataset (i.e., reference data) for genetic discrimination and assignment: *RUBIAS*, which performs genetic stock identification and associated tasks, and *assignPOP*, implementing a supervised machine‐learning genetic‐assignment framework. Using numerical validation analyses, we assessed their suitability and limitations for origin inference at each geographical level. Our results indicate that origin assignment is reliable in *P. pinaster* at the gene pool and region of provenance levels, but less so at the population level, provided that the 10 K SNP markers and a comprehensive genetic baseline are used. Incomplete baselines may result in wrong assignments at any hierarchical level, irrespective of sampling intensity for sampled candidate origins. We provide an extensive and publicly available baseline for *P. pinaster* , offering a useful tool for the management of forest genetic resources of this economically and ecologically important tree species.

## 1 Introduction

Genetic assignment methods can be employed to ascertain population membership of individuals or groups of individuals (Manel et al. 2005), based on the genotypes of the target sample to be identified and those of the candidate sources in a baseline that could contribute to the target sample. Although genetic assignment methods have been most successfully applied to fishery and marine organisms (e.g., Moran and Anderson 2019), most of the studies on forest trees are related to the identification of species, traceability of illegal logging, or custody chain control (Finkeldey et al. 2010; Deguilloux et al. 2003; Finch et al. 2020; Peery et al. 2022; Jolivet and Degen 2012; Degen et al. 2022).

**FIGURE 1:**
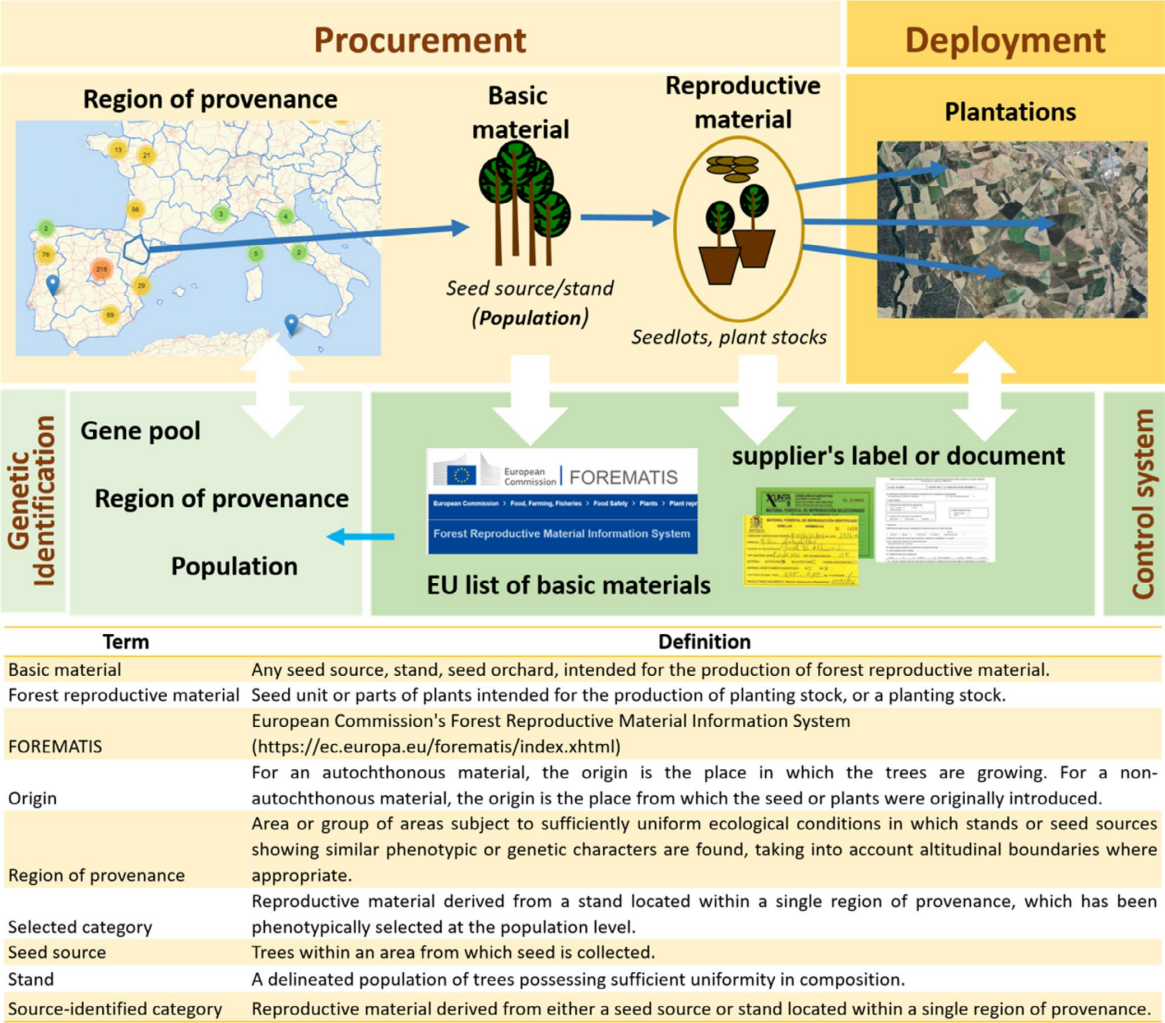
Schematic representation of the process of marketing of *forest reproductive material* (FRM) according to the EU directive (Council Directive 1999/105/EC, 2000) and main objectives of genetic identification of the material along the process. Text provides definitions of the terms according to the EU policy. Qualified and tested materials are not included, as they are mostly based on individual tree characterization.

The origin of *forest reproductive material* (FRM, i.e., fruits, seeds or part of plants intended for the production of planting stock; see glossary in Figure 1) has transcendental implications for the performance and the level of adaptation of forest trees to current and future conditions (Alberto et al. 2013; Leites and Benito Garzón 2023). Thus, it is a major focus of interest in afforestation, forestation, and restoration efforts (Jones 2013; Koskela et al. 2014; Konnert et al. 2015; Jalonen et al. 2018).

There is a long tradition for national and international marketing regulation of FRM (Council Directive 1999/105/EC 2000, Nanson 2001; Nyoka et al. 2011). These regulations aim to protect the end user by preserving the chain of custody throughout the production and marketing process (Figure 1). Reproductive materials are classified according to different categories (e.g., *source identified*, *selected*, *qualified* and *tested*). *Source‐identified* and *selected* categories can be obtained from a large number of approved basic materials in the EU (Alía et al. 2022). For these two categories, reproductive materials shall be characterized, among others, by the *region of provenance* (or *seed zone* in other regulation schemes) and, where appropriate, by the *origin* of the material if known.

The supplier shall inform about the region of provenance of any marketed reproductive material. However, forensic methods may be necessary in order to verify independently the alleged origin or to determine the origin when unknown. For instance, a forensic method for tracing the origin of a seedlot under a given seed regulation scheme involves assigning categorically or probabilistically the lot either to the true region of provenance or to the true basic material from which the seedlot was collected, among all candidate sources (Nanson 2001). The latter approach has been implemented by storing representative samples of all the reproductive material placed on the market at the time of collection from an approved basic material (Finkeldey et al. 2010; Westergren et al. 2017). In view of the cross‐border movement of materials (Jansen et al. 2019), it is crucial to emphasize the importance of sample collection from different countries, which will be used for baseline and genetic assignment.

At a time when extreme weather events and a warming climate are accelerating the decline of European forests (Forest Europe 2015), their restoration with appropriate reproductive material of verifiable origin is more crucial than ever. In cases of uncertainty, supplementing master certificates with unbiased verification methods, such as genetic assignment techniques using molecular markers, can provide an additional layer of reliability. Genetic approaches provide stable, heritable markers that are unaffected by environmental factors; unlike, for example, chemical fingerprinting, making them more reliable for verifying the origin of forest products, ensuring accurate traceability (Beeckman et al. 2020). These genetic approaches rely on statistical methods to estimate the probability of different origins by comparing genotypes in the target sample to expected genotype frequencies in the baseline sources (e.g., Milner et al. 1985; Fournier et al. 1984; Millar 1987; Smouse et al. 1990; Pella and Masuda 2000, 2006).

The power and practical utility of genetic assignment methods is determined by species‐specific factors such as the abundance and spatial distribution of the species, the level and scale of spatial genetic structure, the completeness of the baseline, the size of the target and baseline samples, and the cost. Therefore, even if new genomic resources have the potential to increase the power of genetic assignment (for instance in scenarios with low genetic divergence among candidate sources), it is necessary to evaluate the expected statistical behavior of available methods for a given species, genomic assay, and particular baseline and target samples before practical application. Note that these methods would be applicable to FRM categories source identified and selected (EC Directive 1999/105), but would not be ideal for qualified and tested materials, as these two categories are based mainly on individual selection; therefore, other fingerprinting methods could be used instead (e.g., Cosín‐Roldán et al. 2023 for *Quercus ilex* and *suber*; Olsson et al. 2025 for *Pinus pinea* ).

Forest tree species are characterized by a low level of domestication, and improved material in the early stages of breeding still maintain similar values of diversity and low differentiation with respect to natural populations (Olsson et al. 2023). Studies on the evolutionary genetic diversity and population genetic structure of forest tree species are essential before implementing a tracing method. These studies typically reveal a metapopulation structure, with large‐scale gene pools, reflecting different long‐term evolutionary and demographic histories within species ranges (Milesi et al. 2019; Bruxaux et al. 2024), but also genetic variation within gene pools among distinct geographical or ecological regions (Gugger et al. 2021). At a lower spatial scale, sampling and statistical inference typically focus on populations, which from an evolutionary perspective can be defined as a group of individuals living in close enough proximity that any member of the group can potentially mate with any other member (Waples and Gaggiotti 2006). The degree of genetic differentiation observed at the different hierarchical levels (e.g., among gene pools, among region of provenances and among populations) will determine at which of them genetic assignment is feasible.

*Pinus pinaster* Ait. (maritime pine) is a good example to address the potential practical utility of genetic assignment methods to trace the origin of reproductive material. It is a wind‐dispersed and wind‐pollinated conifer native to the western Mediterranean Basin that has been used extensively in plantations, with a large number of approved basic materials (334 source‐*identified* and 210 *selected* according to FOREMATIS, https://ec.europa.eu/forematis/index.xhtml). Each of the defined regions of provenance belongs to a single gene pool (except FR700, see below); but as largely consistent studies using different kinds of molecular markers show, highly divergent gene pools and large regional and among‐population variation exist (Bucci et al. 2007; Rodríguez‐Quilón et al. 2015; Jaramillo‐Correa et al. 2015; Theraroz et al. 2024).

In this study, we assess the expected feasibility and illustrate the application of two commonly used genetic assignment tools to ascertain the origin of *P. pinaster* individuals at the gene pool, region of provenance, and population levels, given our baseline samples. We provide and evaluate for the first time a range‐wide baseline genotypic dataset for a commercially important conifer species, consisting of 1579 *P. pinaster* trees from 86 populations in 45 regions of provenances and 10 gene pools, genotyped at 10,185 SNPs. We evaluate the reliability of this baseline for genetic assignment based on self‐assignment tests, simulated mixtures of genotypes, and cross‐validation with resampling.

## 2 Material and Methods

### 2.1 Baseline Samples

**FIGURE 2:**
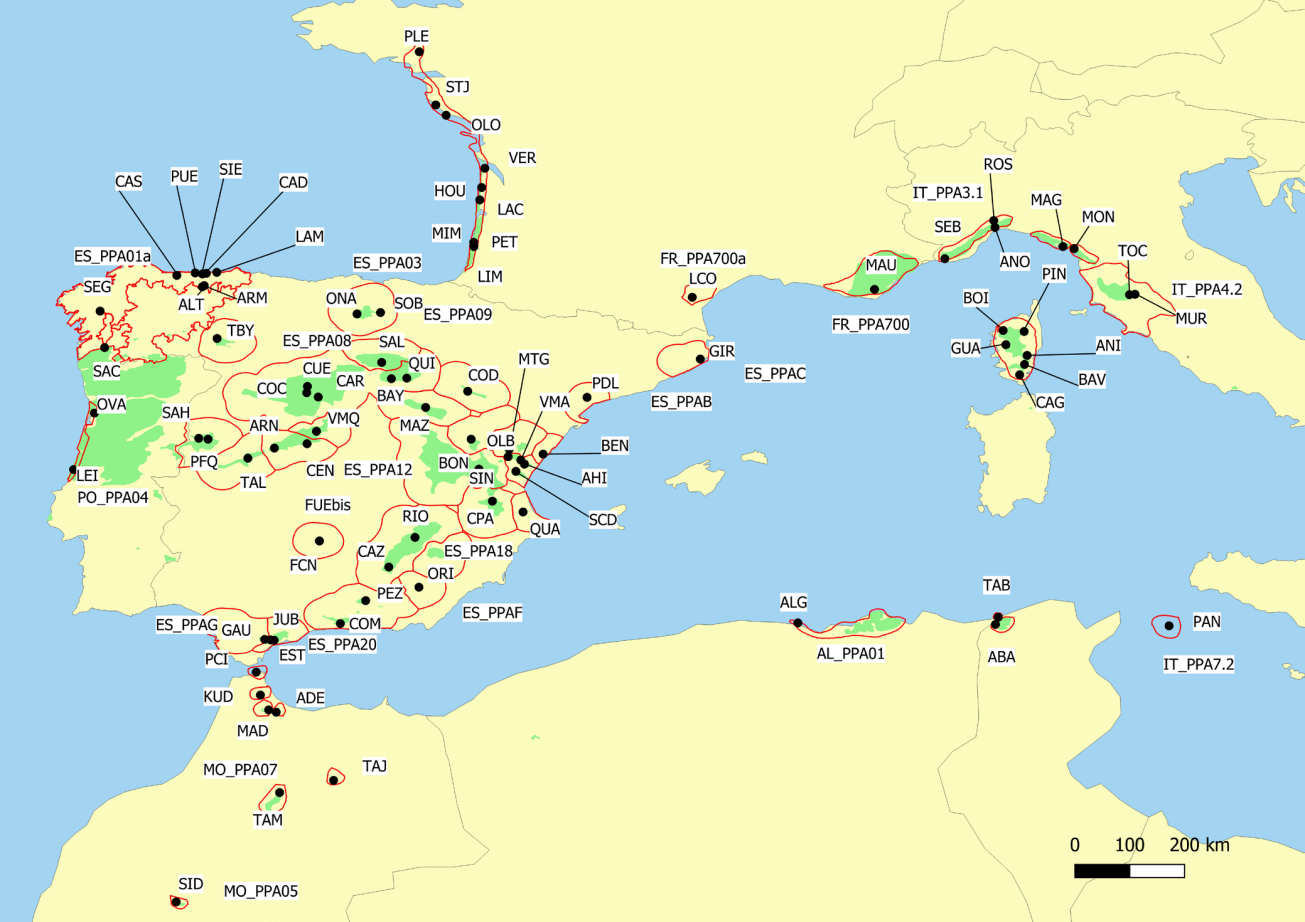
Location of the 86 populations used in the study (dots), 44 regions of provenance (red lines) and known native distribution of the species (green area), obtained from Theraroz et al. (2024). See Table S1 for population details. Approximate distribution of the regions of provenances was obtained from national sources.

We used a baseline dataset of 1579 *P. pinaster* individuals from 86 populations of known origin covering the distribution of the species (Figure 2 and Table S1). The genotypic data that constitutes the baseline of *P. pinaster* is available at Zenodo with DOI: 10.5281/zenodo.14950394. Population in this study refers to a sampling location, usually spreading over a few hectares. These samples comprised 1481 individuals and 82 populations analyzed by Theraroz et al. (2024), complemented by new sampling from two populations in poorly represented areas in Tunisia (25 individuals) and Algeria (22 individuals), a commercial seed lot from Ovar (Portugal) originating from local natural sources (22 individuals), as well as a relict natural stand (29 individuals) growing close to a plantation in Fuencaliente (Central Spain).

### 2.2 Genotyping and Quality Check

DNA was extracted from the samples collected for this study using NucleoSpin Plant II Kit (Macherey‐Nagel GmbH & Co. KG, Düren, Germany), except the samples from Fuencaliente, which were retrieved from Unger et al. (2016). The samples were genotyped using Axiom's 4TREE array (including 13,408 SNPs for *P. pinaster* ) at Thermo Fisher's Microarray Research Services Laboratory, Santa Clara (California, USA). We then merged the newly obtained genotypes with the data set from Theraroz et al. (2024) after retrieving the same SNPs using Affymetrix's software Axiom Analysis Suite v3.1 (Thermo Fischer Scientific, Waltham, MA, USA).

We identified genetic duplicates accounting for genotyping errors using the function mlg.filter from R‐package *poppr* v2.9.5 (Kamvar et al. 2014, 2015), applying the farthest neighbor clustering algorithm, Nei's distance, and a threshold selected with the associated function *cutoff_predictor*, which searches for the best cutoff threshold from statistics obtained using the wrapper function *filter_stats*. The *matchy_pairs* function from R‐package *RUBIAS* v0.3.3 (Moran and Anderson 2019) yielded the same duplicates when setting the minimum fraction of shared non‐missing genotypes at 0.85 and the minimum fraction of matching non‐missing genotypes at 0.97.

We conducted a principal component analysis (PCA) to detect possible outliers, using the R package *snpR* with default settings (Hemstrom and Jones 2022). We excluded individuals identified as probable genetic duplicates and outliers from the baseline dataset.

### 2.3 Genetic Characterization of the Baseline Dataset

We considered three nested levels for analysis: gene pool, region of provenance, and population. First, we located each population to its corresponding region of provenance, according to the division in each country obtained from national sources (see Figure 1). The allocation of populations into gene pools followed Theraroz et al. (2024), which we tested and applied to the new genotyped populations. One of the two populations within region of provenance FR700 (Mediterranean region in France) is known to belong to the French‐Atlantic gene pool (LCO, see Theraroz et al. 2024), so it was assigned to a virtual region FR700a to avoid mixing two gene pools in the same region of provenance. In all, there were 10 gene pools and 46 regions of provenance (Table S1). To estimate how discrete the defined gene pools are, we inferred and visualized individual admixture coefficients using sparse Non‐Negative Matrix Factorization algorithms. For this, we first selected the best *K*‐value (*K* = 10) based on AIC using the fast likelihood approach of Beugin et al. (2018), as implemented in function snapclust.choose.k of the *adegenet* R package (Jombart and Ahmed 2011). We then used the sNMF function from the R package *LEA* (Frichot et al. 2014) implemented in the *snpR* R package (Hemstrom and Jones 2022), considering *K* values ranging from 2 to 10 and running 10 repetitions per *K* value. The results were collapsed into consensus plots using CLUMPP (Jakobsson and Rosenberg 2007). The *pophelper* (Francis 2017) R package was used to visualize the results.

We computed allele‐sharing summary statistics, including hierarchical *F*‐statistics for the three nested levels, with 1000 bootstraps over loci to obtain confidence intervals, and pairwise *F*_ST_ following Weir and Cockerham (1984), using the R package *hierfstat* (Goudet 2005). Population‐specific *F*_ST_ values were calculated with Bayescan v2.1 (Foll and Gaggiotti 2008).

### 2.4 Assignment Methods and Validation Algorithms

We considered two different genetic assignment methods, implemented respectively in R packages *RUBIAS* (Moran and Anderson 2019) and *assignPOP* (Chen et al. 2018). Our goal was not to conduct a formal comparison of the performance of the methods, but rather to assess the expected feasibility and illustrate the application of two readily available genetic assignment tools to trace the origin of *P. pinaster* reproductive material, given our baseline samples. *RUBIAS* incorporates the latest developments in genetic stock identification (GSI) methods, formulated to conduct individual assignments and to obtain unbiased estimates of the proportions of samples in a target mixture (e.g., seed lots) originating from different candidate sources (e.g., basic materials), based on expected genotypic frequencies calculated from baseline samples. *assignPOP* represents a machine‐learning alternative that uses supervised classification functions to build predictive models from genetic (and/or non‐genetic) markers in baseline samples, models that are subsequently used to determine membership probabilities and assign individuals to the source population (basic material in our case) with greatest probability. Both *RUBIAS* and *assignPOP* have built‐in functions that allow evaluating the expected accuracy (proportion of correct assignments) of the methods, which we used to validate our baseline samples at three hierarchical levels: gene pool, regions of provenance, and populations, as described below.

In the case of *RUBIAS*, we carried out the two validation approaches implemented in the available software. The first one is a *cross‐validation leave‐one‐out procedure based on self‐assignments* (*self_assign* function), where each individual in the reference baseline is sampled in turn, and the proportion of correctly assigned genotypes back to their own reference baseline source is used to assess the expected assignment accuracy, given the baseline and assuming that all the possible origins are sampled (see Anderson et al. 2008 for details). *RUBIAS* allows only two hierarchical levels for baseline samples, so we used the *self_assign* function for two separate analyses, first considering regions of provenance nested within gene pools, and second considering populations nested within regions of provenance. In the first case, we assigned each individual to the region of provenance with the highest posterior probability and to the gene pool with the highest sum of posterior probabilities across the regions of provenance within it. We proceeded analogously in the second case, but according to the population with the highest posterior and the region of provenance with the highest sum of posteriors across the populations within it. The second validation approach is based on *Monte Carlo simulation of mixtures of genotypes* from baseline samples, and we characterized the accuracy of assignments into populations, regions of provenance, or gene pools using the *RUBIAS* function *assess_reference_loo* to simulate samples of size 200 assumed to originate from a single source, with 100 independent replications. The simulated samples were then analyzed (assuming their origin is unknown) using the baseline samples and a leave‐one‐out approach to compute genotypic likelihoods and the proportion of individuals correctly assigned to the true source (Moran and Anderson 2019). We thus mimicked a hypothetical practical scenario where a seed lot has been collected from a single unknown source (population, provenance or gene pool) and the ascertainment of its origin is attempted based on a sample of 200 seeds. In practice, according to the EU directive (see Figure 1), seedlots might be a mixture from more than one population, but we did not evaluate this scenario here. In the visualization of the simulation results, we considered the threshold of 90% assignment probability to be acceptable, in line with common practice (Beachman et al. 2020).

In the case of *assignPOP*, we evaluated assignment accuracy using the built‐in *Monte Carlo procedure for cross‐validation* via *resampling* (function *assign.MC*), which randomly samples an adjustable proportion of individuals from each source to be used as a training set, with the remaining being allocated to a test set. In order to assess which genetic markers are more informative, the program also allows choosing subsets of loci with the highest *F*_ST_ values. We used the support vector machine algorithm as a classification model and calculated accuracy separately for assignments to gene pools, regions of provenance, and populations, considering 90% randomly sampled individuals as a training set, 100% of used loci, and 100 independent replicates. In order to test the sensitivity to sample size, we focused on assignment to provenance regions and considered three proportions of training individuals (50%, 70% and 90% from each source) and four proportions of training loci (10%, 25%, 50% and 100% of loci with highest *F*_ST_), with 100 independent replicates for each combination of training proportions.

Additionally, we tested the robustness of *RUBIAS* in terms of false positives (wrong assignments) when the true source (e.g., basic material) is not represented in the baseline samples, a potentially frequent scenario in practice. We focused on an illustrative example by removing selected baseline samples around provenance region ES08 in Spain. The trees in ES08 typically show crooked‐stemmed phenotypes, so it might be problematic from a management perspective if FRM collected within this region of provenance gets wrongly assigned (because of incomplete baselines) to another region with more desirable phenotypes, or vice versa. In addition to ES08, the surrounding regions of provenance having more than one sampled population were also considered as potentially unsampled baselines, namely ES03, ES04, and ES06. Assignment was conducted after removing from the baseline set either one of these entire regions of provenance or one of the baseline populations within them.

We performed all analyses using R v.4.2.2 (R Core Team 2020) with the packages mentioned above, as well as *ggplot2* (Wickham 2016) for visualization.

## 3 Results

### 3.1 Genetic Characterization of the Baseline Samples

**FIGURE 3:**
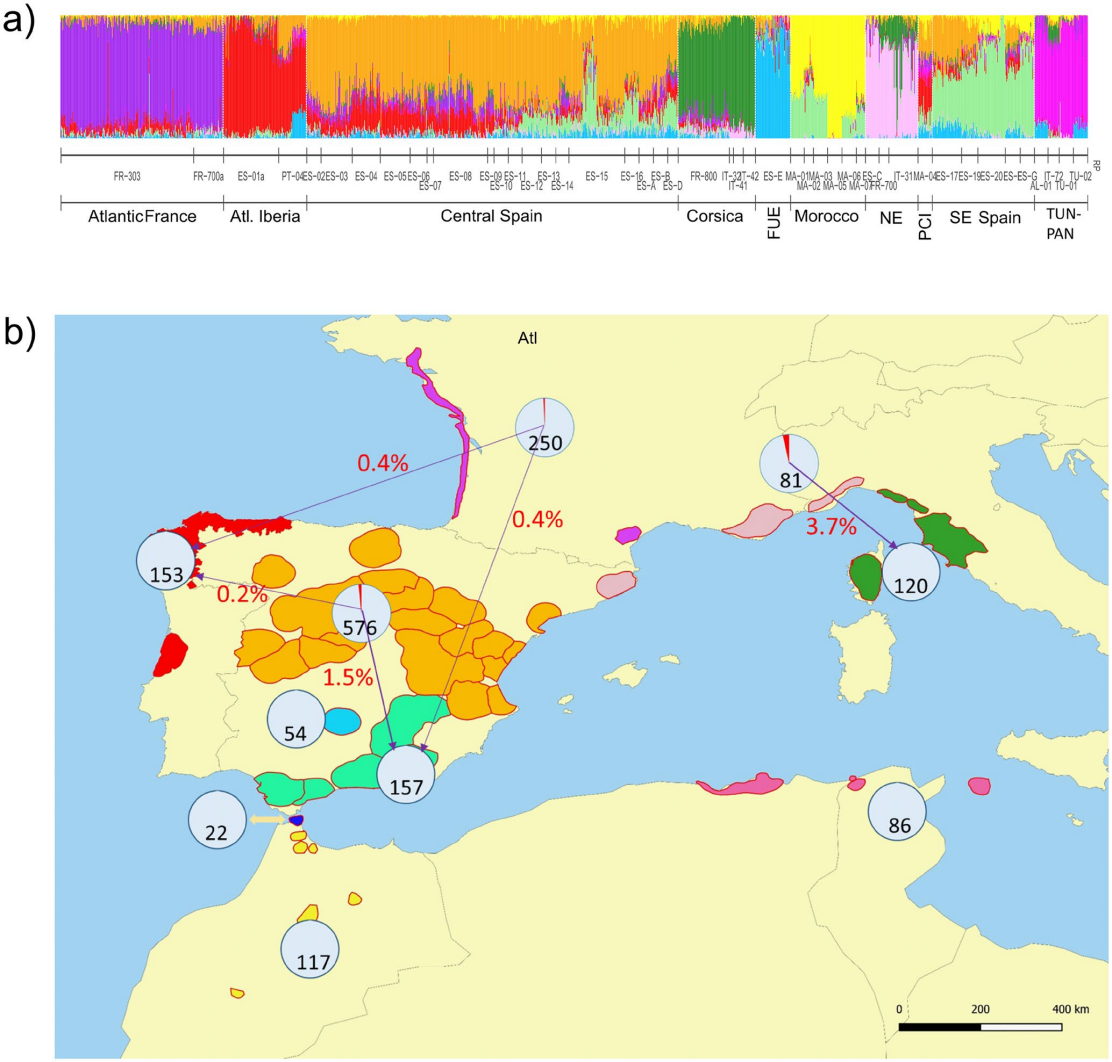
(a) Genetic characterization of 1579 *P. pinaster* individual baseline samples, corresponding to ten predefined gene pools (indicated by the codes in the lower horizontal axis) and 46 regions of provenance (indicated by the codes on the upper horizontal axis). The plot shows individual ancestry coefficients calculated with the sNMF function from R package *LEA* with *K* = 10 groups (indicated by colors), based on 10,185 SNP markers. (b) Expected assignment accuracy to gene pools in *P. pinaster* using *RUBIAS* software. The contours of regions of provenances in each gene pool are depicted. Pie charts indicate the expected proportion of correct (pale grey) and wrong (red) assignments for individuals originating from the corresponding gene pool, according to Monte Carlo simulation of mixtures validation algorithm. Purple arrows indicate wrong gene pool assignments, along with the percentage of wrongly assigned seeds. Baseline sample size is indicated for each gene pool within a circle.

As can be seen from the sNMF analysis and PCA, each region of provenance belongs to a single gene pool, with the exception of FR700 (Figure 3a, Figure S1). Therefore, the initial allocation of populations into gene pools was maintained, and the four new populations were added to the existing gene pools (see Table S1).

The calculated *F*‐statistics showed substantial population genetic structure (*F*_ST_ = 0.276) for *P. pinaster* across its range. The hierarchical analysis indicates that most of the observed genetic differentiation corresponds to divergence among gene pools (*F*_ST_ = 0.137). Although variation among regions of provenance within gene pools is lower but still significant (*F*_ST_ = 0.024), the differentiation among populations within regions of provenance (*F*_ST_ = 0.099) is of a similar magnitude to that observed among gene pools. The results show moderate differentiation among populations within regions of provenance and high variation among individuals within populations. Gene diversities were similar across hierarchical levels, ranging from an average of 0.225 in populations (Table S2) to an average of 0.238 in gene pools.

### 3.2 Expected Assignment Accuracy to Gene Pools

The genetic assignment methods implemented in *RUBIAS* and *assignPOP* behaved similarly well when ascertaining gene pool origin, as indicated by their respective built‐in validation algorithms. Specifically, validation of *RUBIAS* based on Monte Carlo simulation of mixtures of genotypes (Figure 3b) yielded a very high average assignment accuracy to gene pools for simulated 200‐seed lots (99.2% on average, 97.3% for North East gene pool and > 99% for the others, see Figure 3b, Table S3). The few individuals in the simulated seed lots that were assigned to an incorrect gene pool were mostly in gene pools that have a higher level of admixed ancestry and from neighboring gene pools (individuals from Central Spain wrongly assigned to Southeastern Spain, or from North East assigned to Corsica) (Figure 3). Cross‐validation of *RUBIAS* via leave‐one‐out self‐assignments consistently indicated that individual *P. pinaster* assignments are correct on average 99.4% of the time at the gene pool level (Table S4), ranging between 96.3% and 100% depending on the actual gene pool of origin, with the lower accuracies observed for North East (96.3%) and Central Spain (98.3%).

The expected accuracy of machine‐learning‐based assignments to gene pools using *assignPOP* and our baseline samples was similarly high overall (mean 99.4%, range 96.3%–100%), as indicated by cross‐validation via Monte Carlo resampling (Table S5).

### 3.3 Expected Assignment Accuracy to Regions of Provenance

**FIGURE 4:**
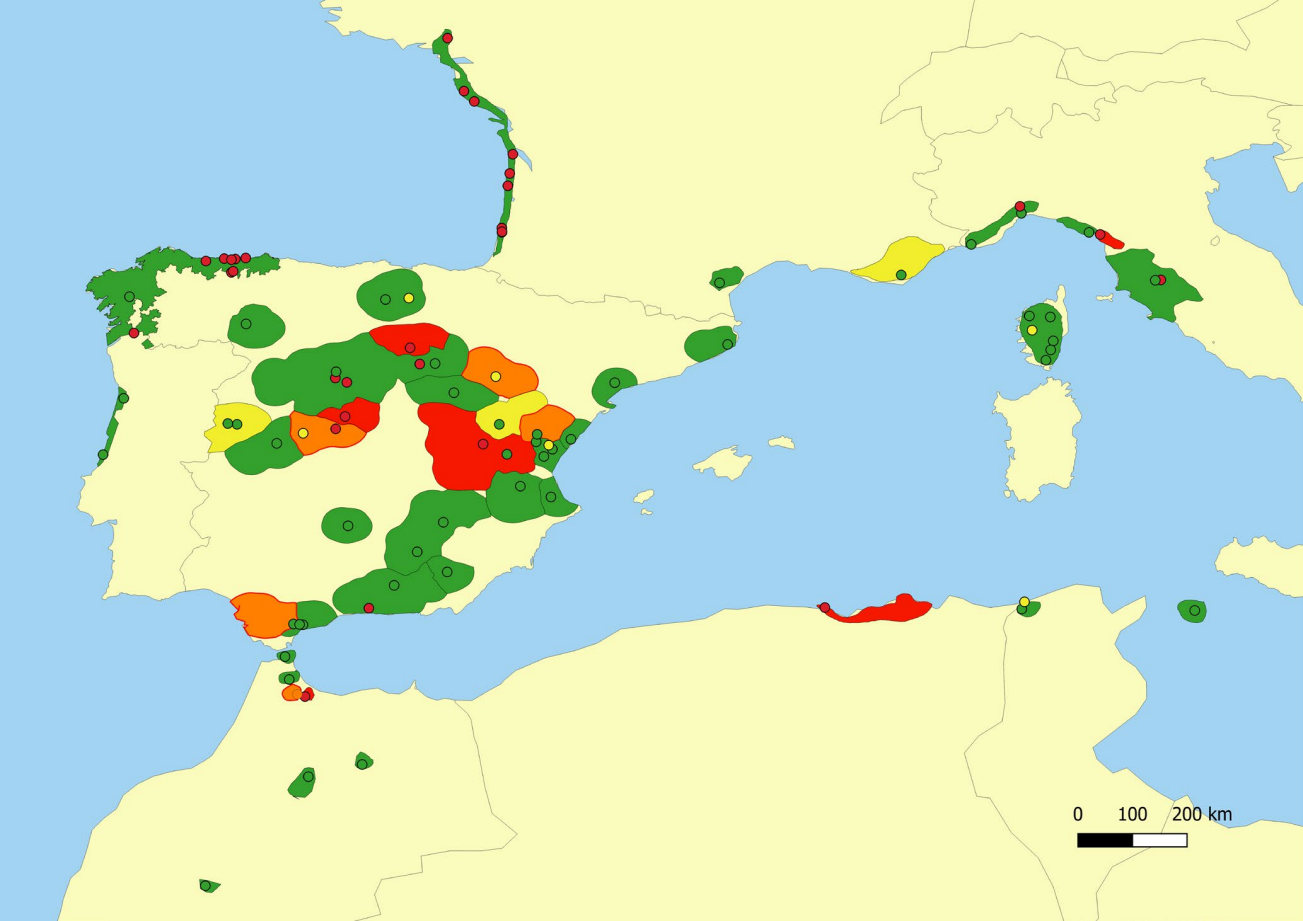
Expected assignment accuracy to regions of provenance (shapes) and populations (circles) in *P. pinaster* using *RUBIAS* software, according to Monte Carlo simulation of mixtures validation algorithm. The color codes for regions of provenances and populations indicate the assignment accuracy: Green > 0.90, yellow 0.80–0.90, orange 0.70–0.80, red < 0.70.

Validation via Monte Carlo simulation of mixtures of genotypes indicated that the expected accuracy of *RUBIAS* for genetic assignments of seed lots to regions of provenance was somewhat lower than to gene pools, but still high (86.1%) on average, remaining above 90% in most cases, with exceptions generally corresponding to regions of provenance comprising a single sampled population and/or with small baseline sample sizes (Figure 4, Table S6). The latter was the case, for instance, of the two populations with the lowest accuracy (0%), namely ES‐09 (all 10 individuals wrongly assigned to ES‐08) and IT‐3.2 (all six individuals wrongly assigned to either IT‐4.2 or IT‐4.1). Cross‐validation of *RUBIAS* via leave‐one‐out self‐assignments yielded a similar expected accuracy for the assignment of individuals to regions of provenance (88.6% on average), and a similar sensitivity to low baseline sample size (Table S7).

The expected accuracy of genetic assignments of individuals to regions of provenance using *assignPOP* was very similar (average of 88.8% across provenances) to that of *RUBIAS*, as indicated by cross‐validation via Monte Carlo resampling, with a similar range between 0 and 100% and the lowest values associated with small baseline samples (Table S5).

### 3.4 Expected Assignment Accuracy to Populations

The expected accuracy of *RUBIAS* for assigning seed lots to populations was lower than for assigning them to regions of provenance, with an average of 73.7% and a range of 0%–100% across populations, according to Monte Carlo validation via simulated mixtures of genotypes (Figure 4; Figure S2). Validation via leave‐one‐out self‐assignments yielded a nearly identical expected accuracy for the assignment of individuals to populations with *RUBIAS* (73.8% on average; Table S2). The accuracy of the assignments ranged from 0% to 100% across populations, generally not reaching 90%. This shows that, when more than one population from the same region of provenance had been genotyped, they could not always be reliably identified as distinct origins (see Table S2). In particular, all individuals from some populations were wrongly assigned, which decreased the average proportion of correct assignments at the population level, while still being mostly assigned to the correct region of provenance. The results of genetic assignment to populations with *assignPOP* had very similar expected accuracy, according to cross‐validation via Monte Carlo resampling, with a mean value of 75.3% and a range between 0% and 100% across populations (Table S5), with minimum and maximum expected accuracies corresponding to the same populations as in the case of assignments with *RUBIAS*.

### 3.5 Sensitivity to Sample Size and Missing Baselines

**FIGURE 5:**
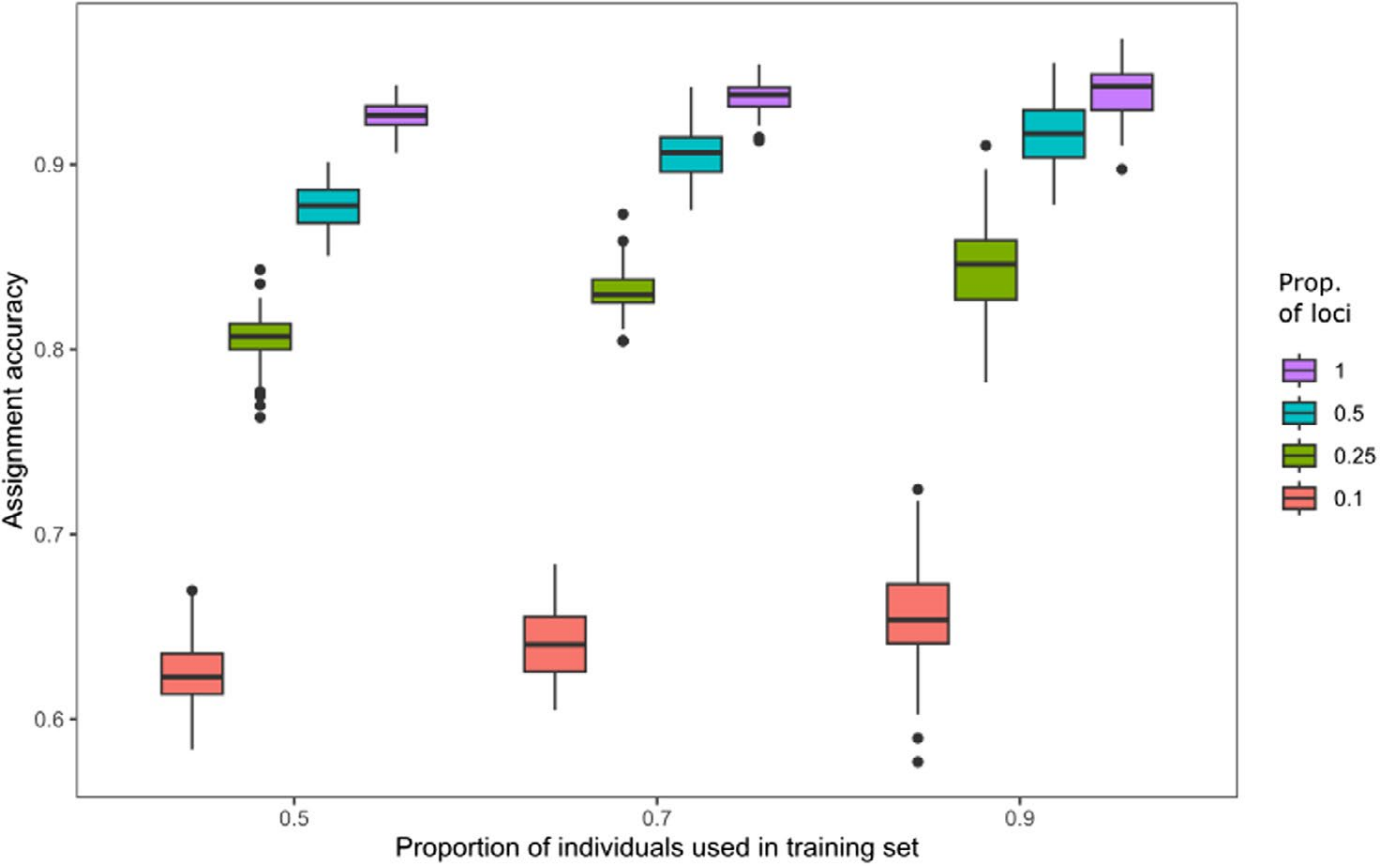
Expected accuracy of the assignment of individuals to regions of provenance using *assignPOP* software with different proportions of individuals in the training set (0.5, 0.7 and 0.9) and different proportions of used loci (0.1, 0.25, 0.5 and all loci). Results are based on cross‐validation via Monte Carlo resampling with 100 replicates for each combination of parameters. The total number of SNP loci was 10,185, which were ranked and subsampled based on their *F*_ST_ values.

The expected accuracy of genetic assignment to regions of provenance using *assignPOP* decreased with diminishing numbers of used loci, whereas the effect of the number of individuals used for training was comparatively smaller (Figure 5). With 0.1 proportion of loci (i.e., approx. 1000 markers) the average of correct assignments to regions of provenance ranged from 0.624 to 0.655 with different proportions of individuals in the training set (0.5, 0.7 and 0.9); with 0.25 proportion of the loci the values ranged from 0.807 to 0.844; with 0.5 from 0.877 to 0.915; and with all loci from 0.926 to 0.940.

Using *RUBIAS* software, removing an entire region of provenance from the reference baseline invariably resulted in tested individuals actually originating from that particular region being wrongly assigned to other (genetically close) regions represented in the baseline (Table 1). In some cases (when either ES04, ES06 or ES08 region of provenance was missing), wrong assignments were distributed across several regions with low or moderate (< 61%) probabilities, while in one case (ES03 missing) individuals were preferentially wrongly assigned to a single region (with probability > 80%). In addition, genetic assignment to provenance regions was variably affected by the true source population not being represented in the baseline when other populations from the source provenance region were (Table 1). In particular, the true source provenance region was still correctly identified as the most likely source (albeit with reduced posterior probability) for provenance regions ES03 and ES08 when the specific source population was missing from the baseline. This was not the case, however, for provenance region ES06 and its two baseline populations, or for provenance region ES04 when the ES04_SAH population was missing.

**TABLE 1:** Genetic assignment of *P. pinaster* individuals to regions of provenance using *RUBIAS* when either the true source region of provenance (codes in normal font) or the true source population (codes in italics) are missing from the reference baseline samples. Estimates are the proportion of individuals in the test set (assumed to have originated from the source that is missing in the baseline) assigned to the specified provenance region. Median, lower (loCI) and upper (hiCI) 95% credibility interval limits are included. Up to three other regions of provenance are included with median values greater than zero.

Missing baseline	Assigned provenance region				Other regions of provenance assigned (median)
	Code	Median	loCI	hiCI	
ES03	ES08	0.813	0.678	0.905	ES04 (0.016), ES06 (0.046), ES11 (0.095)
*ES03_ONA*	ES03	0.971	0.852	0.999	
*ES03_SOB*	ES03	0.807	0.626	0.928	ES08 (0.158)
ES04	ES08	0.339	0.214	0.488	ES05 (0.239), ES06 (0.248), PT04 (0.069)
*ES04_PFQ*	ES04	0.916	0.768	0.987	ES08 (0.036)
*ES04_SAH*	ES05	0.281	0.126	0.496	ES06 (0.207), ES08 (0.166), PT04 (0.138)
ES06	ES05	0.609	0.432	0.771	ES04 (0.055), ES08 (0.264), ES11 (0.024)
*ES06_ARN*	ES05	0.641	0.407	0.842	ES04 (0.041), ES08 (0.241)
*ES06_CEN*	ES05	0.396	0.139	0.701	ES04 (0.074), ES06 (0.350)
ES08	ES05	0.347	0.252	0.45	ES06 (0.143), ES09 (0.209), ES11 (0.178)
*ES08_BAY*	ES08	0.959	0.794	0.999	
*ES08_CAR*	ES08	0.901	0.547	0.997	
*ES08_COC*	ES08	0.905	0.716	0.987	ESF (0.039)
*ES08_CUE*	ES08	0.846	0.999	0.973	
*ES08_QUI*	ES08	0.459	0.849	0.676	ES11 (0.281)

## 4 Discussion

Our validation analyses indicate that in a forest tree species that is highly geographically structured, such as *P. pinaster* (Jaramillo‐Correa et al. 2015), origin identification via genetic assignment methods can be expected to work well at the gene pool and region of provenance levels (but less so at the population level), provided sufficient markers and a comprehensive genetic baseline are used. Assignment accuracy increased with baseline sample size and number of loci, as reported in previous studies (Ackerman et al. 2011; Beacham et al. 2012; Araujo et al. 2014), but incomplete baselines may result in wrong assignments irrespective of the sampling intensity of candidate sources.

### 4.1 Considerations on the Baseline

The baseline presented in this study is the most comprehensive genotypic data set for *P. pinaster* available so far, but still not perfect. In order to achieve reliable assignment, the baseline should be as comprehensive as possible, representing all possible origins at the desired hierarchical level of assignment, with a sufficient number of individuals per population, populations per region of provenance, and regions of provenance per gene pool. Natural populations have been the main sampling target in previous population genetic range‐wide studies of *P. pinaster* (e.g., Bucci et al. 2007; Theraroz et al. 2024); however, in addition to these natural populations, plantations of known origin should be included in the baseline (clearly labeled as such), as they represent a high proportion of the species distribution area (Freer‐Smith et al. 2019). These plantations should not be discarded a priori as candidate sources of FRM of unknown origin. They may affect the spatial genetic structure of the species in the long term and may have particular phenotypic characteristics as a result of accelerated “landrace‐like” development in plantations (Ribeiro et al. 2001; MacLachlan et al. 2021; Olsson et al. 2023).

Our baseline includes all previously defined gene pools for the study species, and it comprises abundant population replication across most of them, except for those located in very small areas (e.g., Fuencaliente, Punta Cires and the Tunisian populations), in which sampling cannot be significantly extended. Within gene pools, sample sizes for different regions of provenance were unequal and should be improved, especially those with still a low level of accuracy (e.g., ES‐PPA07 or ES‐PPA09). Furthermore, a few of the defined regions of provenance of the species are not represented in our baseline (e.g., regions of provenance consisting of plantations in Portugal were not included), but could be easily added in the future to avoid wrong assignments when testing samples potentially originating from those regions. Constructing a comprehensive baseline at the population level is far more challenging for widespread species such as *P. pinaster* , however, due to the large amount and continuous distribution of existing populations. Therefore, results of genetic assignment at the population level using the current baseline should be taken with caution. Likewise, the large amount of some categories of FRM available for many species (e.g., 534 basic material units of the *source‐identified* and *selected* categories are included in the European FOREMATIS database for *P. pinaster* ) does not allow a cost‐effective genetic characterization of those materials exhaustive enough for accurate genetic assignment in most species and countries.

The dataset made available in this study represents the first attempt to build a range‐wide baseline for genetic assignment of FRM in a forest tree species, and it should be regarded as the basis for a comprehensive reference database for origin identification, to be complemented with new data for *P. pinaster* and to be extended to other important forest tree species. It should be noted, however, that accurate genetic assignment will be more challenging for species with weaker genetic differentiation and/or more continuous spatial distributions than *P. pinaster* , as illustrated, for instance, by the decreased assignment accuracy that we observed in our analysis for provenance regions with low genetic differentiation from other provenances.

### 4.2 Expected Assignment Accuracy

According to their built‐in validation algorithms, the genetic assignment methods implemented in *RUBIAS* and *assignPOP* show very similar expected accuracies across all hierarchical levels considered for our *P. pinaster* baseline (gene pool, region of provenance and population), and both could be recommended as tools for FRM identification. *RUBIAS* conducts individual assignments and additionally explicitly estimates mixture proportions, while it also conveniently implements assignment at two hierarchical levels (e.g., populations nested within regions of provenance), and it is quite flexible to summarize statistics. This method has long been used in fisheries research and management for genetic stock identification and is still frequently updated (Anderson et al. 2008; Beachman et al. 2020; Moran and Anderson 2019). Our study shows that it is promising to extend its practical application to forestry.

Our results also showed that if the true origin is absent from baseline samples, then most test individuals will be assigned to an incorrect sampled baseline, typically to the one genetically closer to the true origin, sometimes with high probability (Beacham et al. 2012). The genetic assignment method should be able to detect cases where the actual source of the material is not an approved unit. Such cases could constitute fraud in the marketing of FRM by violating EU regulations, and in addition, there is a risk of wrongly assigning the material to an approved basic material—especially if the actual source is not included in the reference dataset used for testing. In addition, and maybe more surprisingly, our results showed that missing a single population within a region of provenance may result not only in higher uncertainty, but even in wrong assignments to a different region. The reason could be related to the small sample size of the remaining sampled population within the region (e.g., ARN and CEN from ES06), and/or to high genetic differentiation among populations within some regions (e.g., PFQ and SAH from ES04). Several approaches have been proposed to address the challenging task of detecting individuals originating from unsampled sources (e.g., Smouse et al. 1990; Dawson and Belkhir 2021; Pella and Masuda 2006). Among them, the *RUBIAS* software manual (see https://github.com/eriqande/rubias) proposes the usage of *z*‐scores for raw genotype log‐likelihoods, comparing their value for each test individual (calculated based on the mean and standard deviation of genotype log‐likelihoods of reference individuals in the candidate source with the highest posterior probability) against the standard normal distribution. However, to our knowledge, these approaches have not been yet properly validated and deserve further theoretical examination before practical application.

Moreover, our analyses indicated that assignment accuracy increases with baseline sample size, number of loci, and genetic divergence among candidate sources. Provided a large number of molecular markers (around 10,000 in our baseline), it is possible to achieve good assignment accuracy for *P. pinaster* at the gene pool and region of provenance levels. By contrast, the smaller baseline sample sizes and the low level of divergence among *P. pinaster* populations within some particular regions make assignment to populations more difficult, because the number of markers and sample size needed to discriminate candidate sources is inversely proportional to their genetic divergence (Patterson et al. 2006). As assignment accuracy is very high at the gene pool level, fewer markers would suffice to distinguish between gene pools. However, more markers are required for genetic assignment than for verifying a suspected origin (i.e., the probability of exclusion), where very high probabilities can be achieved with a small number of markers (Primmer et al. 2000).

Given the strong macro‐geographical genetic structure of *P. pinaster* , the assignment accuracies to each of the defined gene pools was, as expected, very high, with a very small proportion of individuals incorrectly assigned to adjacent gene pools. The wrong assignments could be due to occasional gene flow between the gene pools. Accurate identification of individual origin at this geographical level could be of major interest for conservation, as gene pool limits may be considered a template for designing management units (MUs) sensu Moritz (1994) within the natural range of *P. pinaster* (Bucci et al. 2007; Rodríguez‐Quilon et al. 2016). Accurate assignment to gene pools is also most useful in the context of marketing of forest products, allowing, for instance, an efficient detection of unwanted Iberian FRM of *P. pinaster* marketed within France (as conducted with more geographically limited baselines using cpSSRs by Ribeiro et al. 2002), or range‐wide timber geographical origin identification (as conducted with cpDNA in other species, e.g., Blanc‐Jolivet et al. 2018, Ng et al. 2017, Deguilloux et al. 2003).

At lower geographical scale, most (around 70%) of the regions of provenance showed high (> 90%) assignment accuracy. These results suggest that the assignment methods employed provide a useful molecular tool for verifying the declared provenance region of *P. pinaster* FRM used for afforestation (although applicable only in some gene pools), which is considered challenging for many forest tree species (Finkeldey et al. 2010; Peery et al. 2022). Some of the regions with poor assignment accuracy had very few sampled individuals (ES‐PPA09 and IT‐PPA3.2), stressing the importance of collecting large enough baseline samples, which would be easily achievable for these provenances. For some other regions of provenance, however, reaching high assignment accuracy may be difficult even with many markers and sampled individuals because of very low genetic differentiation from other provenances (e.g., the pair of provenances ESPPA10 and ESPPA11 with a pairwise *F*_ST_ 0.038, and the trio ESPPA12, ESPPA13 and ESPPA14 with pairwise *F*_ST_ values ranging from 0.031 to 0.044). Low discrimination among particular provenances impinges on the application of assignment methods for the identification of marketed FRM (EU Directive 1999/105), as it would compromise the detection of allochthonous material translocations. It should be noted, however, that poorly differentiated provenances within the same gene pool are typically geographically proximate, morphologically similar, and exhibit only minor climatic differences (Alia and Moro 1996). If low molecular differentiation among these provenance regions reflects low adaptive divergence, there would be no practical need for the end user to distinguish between them genetically. Overall assignment accuracy for the species could be improved by pooling these provenances. An alternative approach would be to attempt to improve molecular discrimination by using newly developed markers (e.g., Hufford et al. 2016; Fremount et al. 2021).

At the population level, assignment accuracy was especially low for populations with smaller sample sizes, or when they were located within particular regions of provenance where several genetically similar populations had been included in the baseline. Population sample size and population‐specific genetic differentiation (population‐specific *F*_ST_ values) were in fact both significantly correlated with the observed population assignment accuracy values, explaining about 37% of its variance (multiple linear regression *R* ^2^ = 0.366, *F =* 23.98, *p* < 0.001; unique variance estimates of 33% and 6% for sample size and *F*_ST_, respectively). Populations with higher assignment accuracy frequently were the only ones sampled within some provenance regions, and therefore corresponded to cases where the population and region of provenance effects were confounded, in absence of population sampling replication. Some isolated and small baseline populations (e.g., in Fuencaliente, southeastern Spain, North Africa and Pantelleria) are actually the only ones present in the region. In other cases, sampling more than one population within the corresponding region of provenance would probably result in improved assignment accuracy to that region but decreased accuracy at the population level. From a practical point of view, we cannot recommend the used methods for accurate determination of the population origin of FRM (e.g., identification of the basic material or conservation unit from which a seed lot was collected), unless baseline sample sizes were increased at the population level and yielded higher expected accuracies in validation tests as the ones conducted here.

The application of forensic methods, including DNA fingerprinting, to forest tree species has been widely implemented for ascertaining the origin of timber products (e.g., oak barrels, illegal timber) (Deguilloux et al. 2003; Ng et al. 2017; Finch et al. 2020), but this study demonstrates their application, based on a well‐documented baseline, to the identification of FRM in restoration and conservation activities. The proposed baseline and assignment approaches could be easily applied to the analysis of *P. pinaster* seedlots and planting stock, or to determine the source of existing plantations of unknown origin, helping to understand complex patterns of genetic structure in some of the regions where undocumented historical seed transfers have been frequent (e.g., Portugal and Northern Spain). The proposed framework for origin identification can also help in landscape management within regions consisting of a mosaic of natural forests and plantations, where no records on the origin of the stands are available. This information could help update maps of natural forest distribution, which are essential to the sustainable management of biodiversity. The baseline used in this study was generated with a commercially available genotyping array, ensuring robust and accessible data that can easily be complemented with new data or be used as reference for other studies. This accessibility enhances the applicability of our research to various real‐life scenarios.

## Conflicts of Interest

The authors declare no conflicts of interest.

## Supporting information


**Figure S1:** eva70145‐sup‐0001‐Figures.pdf.

**Table S1:** eva70145‐sup‐0002‐Tables.zip.

## Data Availability

Data for this study are available at Zenodo with DOI: https://doi.org/10.5281/zenodo.14950394 (to be publicly released after manuscript is accepted for publication).
