# Supplementary material for: Genetic Assignment at Different Geographical Levels: A Case Study in a Forest Tree Species (Pinus pinaster Ait.) Using SNP Markers
Source: Evol Appl. 2025 Dec 2;18(12):e70145. doi: 10.1111/eva.70145 (PMC12670294; doi:10.1111/eva.70145)

**Supplementary Figure S1.** A) Population genetic structure of maritime pine genetic baseline dataset of 1,579 individuals, based on 10,185 SNP markers. Individual ancestry coefficients at K ranging from 2 to 10 (optimal K=10) estimated with the sNMF function from R package *LEA*. Individuals are grouped per corresponding regions of provenances (alphanumerical codes in the x-axis) and gene pools (lower horizontal bar).

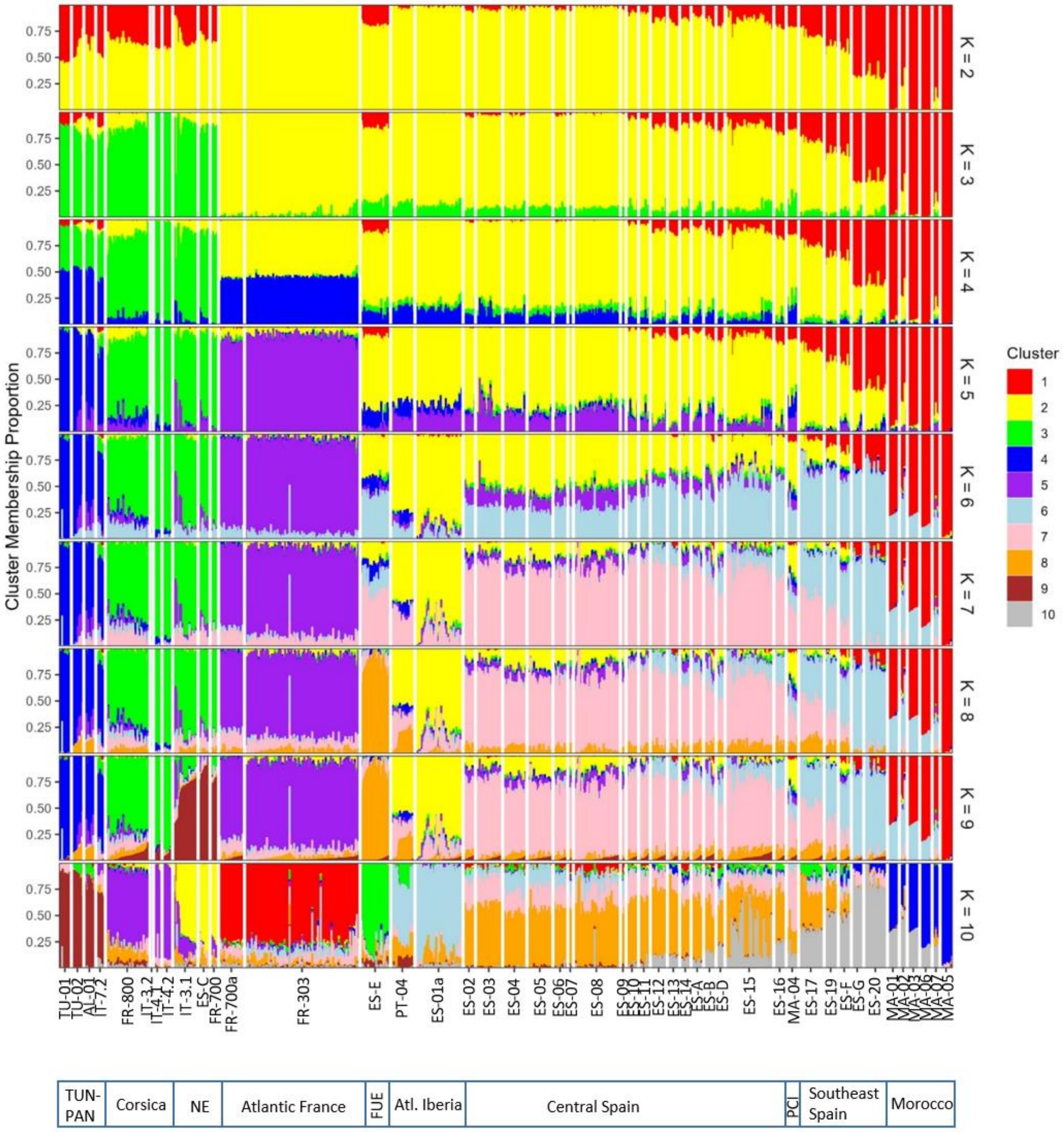

B) A smartPCA calculated using the R package *snpR* (Hemstrom and Jones, 2022). Individuals colored by corresponding gene pool (note that color codes are independent of those in panel A).

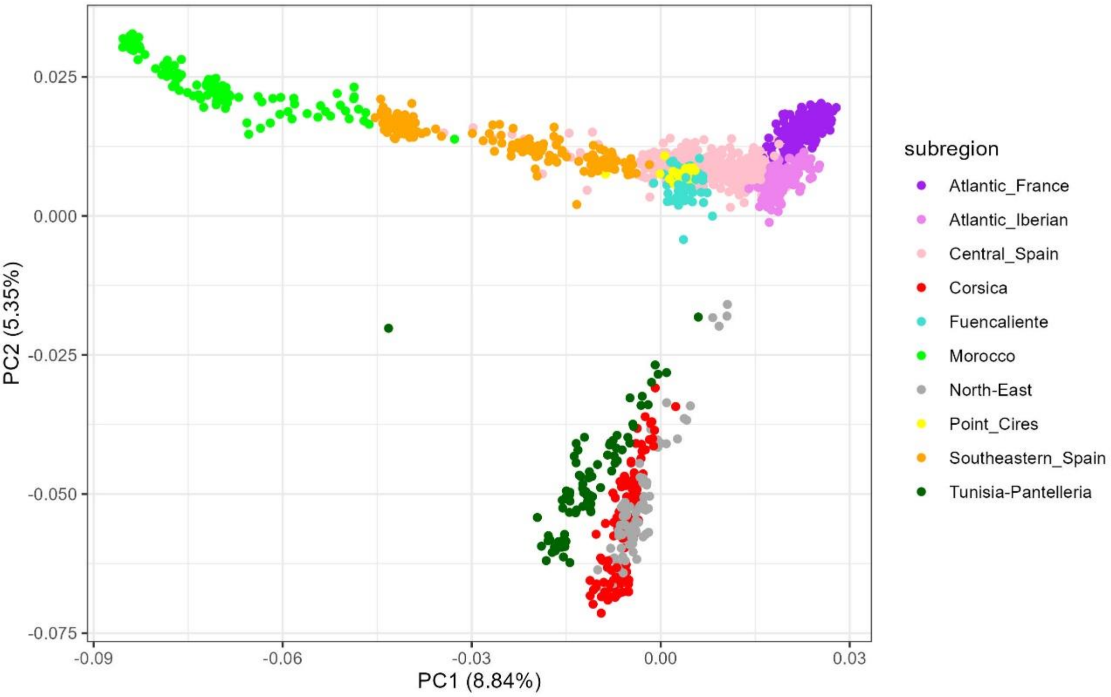

**Supplementary Figure S2.** A) The average percent accuracy for estimated seed lot compositions for individual populations in a region of provenance (RP) back to population (open portion of bar) and to RP (gray portion of bar) for all populations in a RP, with the number of populations in the RP reported to the right of the bar. A 90% accuracy level is indicated in the figure with a dash line. The simulation results were visualized as barplots with the help of scripts provided by Ben Sutherland via Github (https://github.com/bensutherland/simple_pop_stats/tree/master").

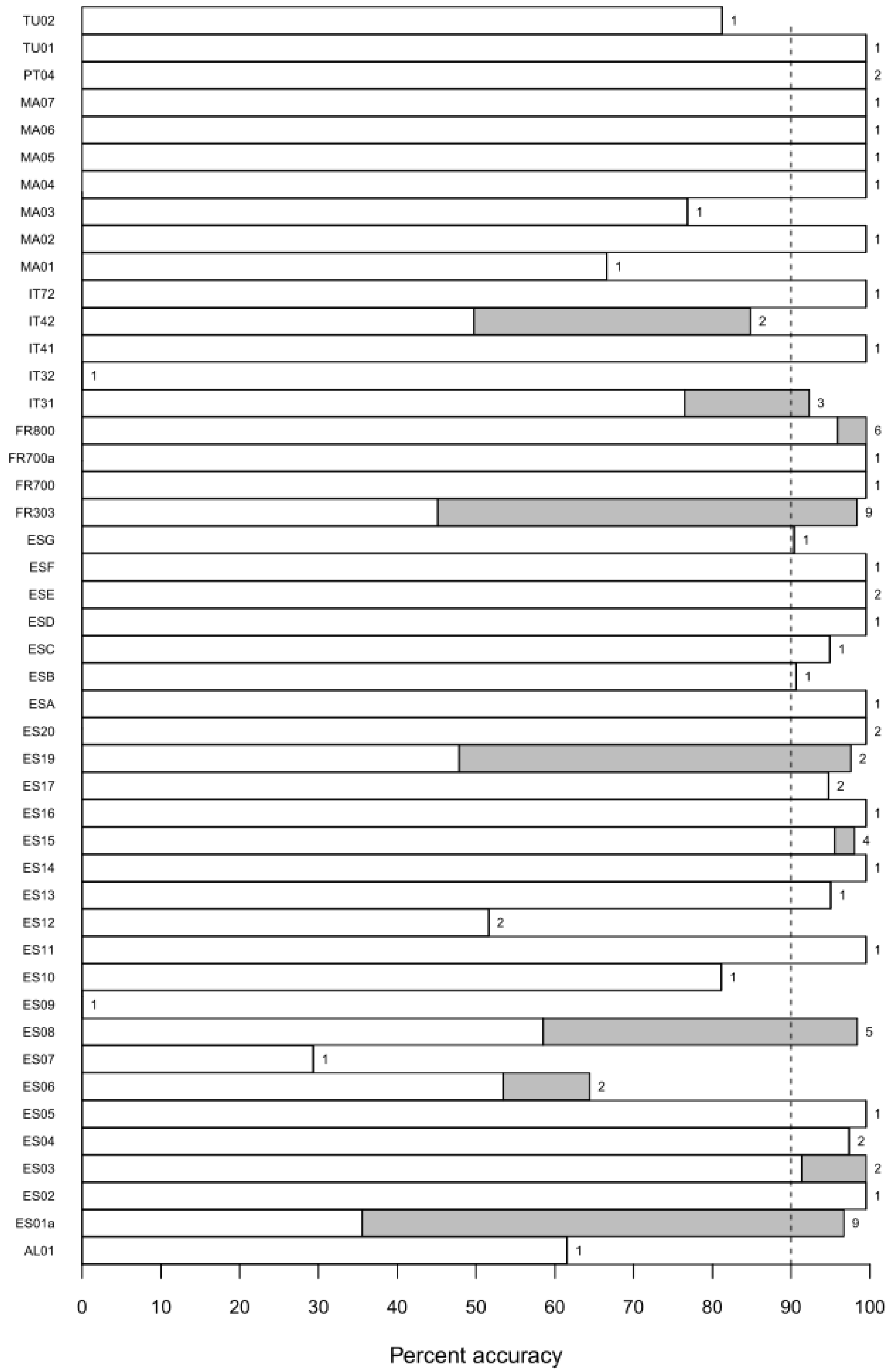

B) The average percent accuracy for estimated seed lot compositions for individual populations in a region of provenance (RP). The color codes indicate the assignment accuracy: green >0.90, yellow 0.80 – 0.90, orange 0.70 – 0.80, red < 0.70. Codes for region of provenances are shown.

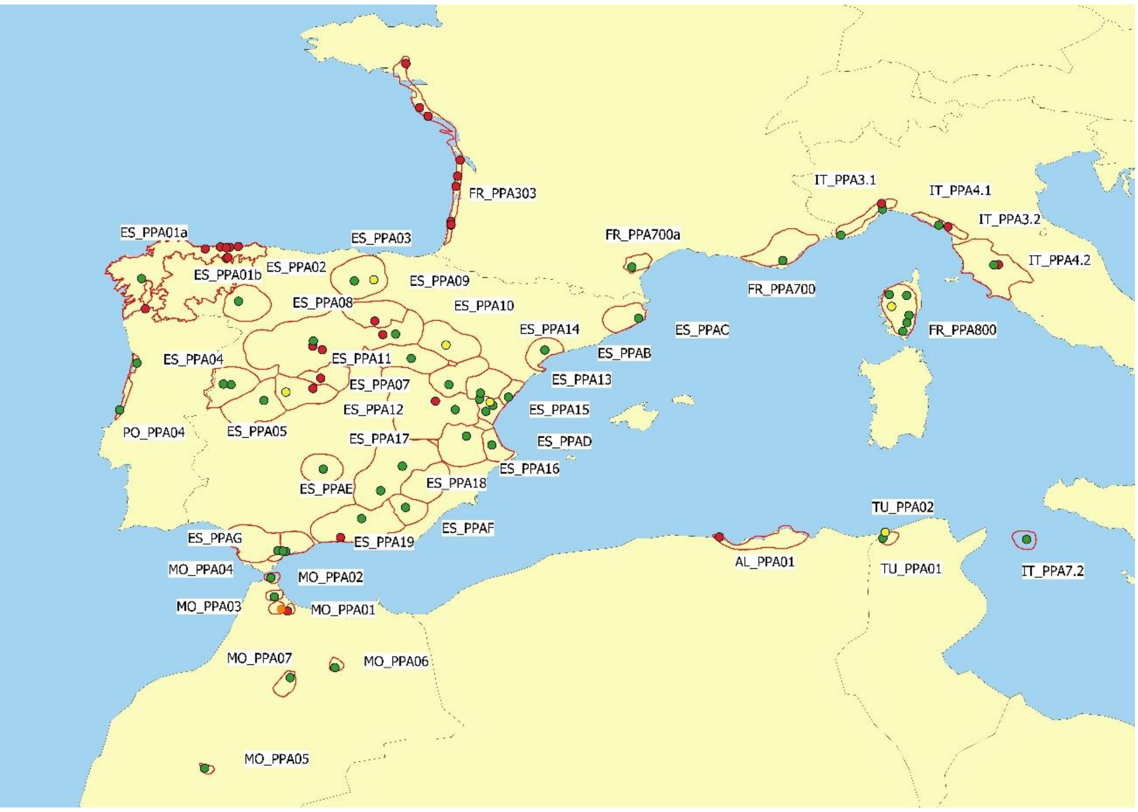

Supplement: Supplementary file 1 — Figure S1: eva70145‐sup‐0001‐Figures.pdf. [file EVA-18-e70145-s002.pdf]
