# Supplementary material for: Genetic Assignment at Different Geographical Levels: A Case Study in a Forest Tree Species (Pinus pinaster Ait.) Using SNP Markers
Source: Evol Appl. 2025 Dec 2;18(12):e70145. doi: 10.1111/eva.70145 (PMC12670294; doi:10.1111/eva.70145)
Supplement: Supplementary file 2 — Table S1: eva70145‐sup‐0002‐Tables.zip. [file EVA-18-e70145-s001.zip › eva70145-sup-0002-TableS1-S7@Supplementary Tables_v2.pdf]

Supplementary Table S1. Baseline dataset of 1,579 *Pinus pinaster* individuals used in the study. The population, number of individuals per population (N), coordinates, country, provenance region, gene pool and sample type are indicated. The populations in bold were genotyped for this study while the others were obtained from Theraroz et al. (2024).

Gene pool	Region of provenance	Population ID	N	Population name	Country	Latitude	Longitude	Altitude	Type
Atlantic France	FR-303	HOU	25	Hourtin	FRA	45.1833333	-1.15	28	Juvenile needles
Atlantic France	FR-303	LAC	21	F.D. de Lacanau	FRA	44.946	-1.187	36	Adult needles
Atlantic France	FR-700a	LCO	46	Les Corbières	FRA	43.082	2.878	216	Adult needles
Atlantic France	FR-303	LIM	25	F.D. de Lit et Mixe	FRA	44.05226	-1.300667	45	Adult needles
Atlantic France	FR-303	MIM	18	Mimizan	FRA	44.1341667	-1.30316667	18	Juvenile needles
Atlantic France	FR-303	OLO	24	Olonne sur Mer	FRA	46.5659167	-1.83061111	12	Juvenile needles
Atlantic France	FR-303	PET	22	Petrocq	FRA	44.0639722	-1.29955556	21	Juvenile needles
Atlantic France	FR-303	PLE	19	Pleucadec	FRA	47.7811944	-2.34366667	70	Juvenile needles
Atlantic France	FR-303	STJ	25	St-Jean des Monts	FRA	46.7639167	-2.02863889	6	Juvenile needles
Atlantic France	FR-303	VER	25	Le Verdon	FRA	45.5519444	-1.09055556	10	Juvenile needles
Atlantic Iberian peninsula	ES-01a	ALT	8	Alto de la Llama	ESP	43.2834235	-6.49427025	526	Juvenile needles
Atlantic Iberian Peninsula	ES-01a	ARM	8	Armayán	ESP	43.3048022	-6.4582725	559	Juvenile needles
Atlantic Iberian Peninsula	ES-01a	CAD	8	Cadavedo	ESP	43.5399652	-6.41784706	164	Juvenile needles
Atlantic Iberian Peninsula	ES-01a	CAS	8	Castropol	ESP	43.500783	-6.98250814	158	Juvenile needles
Atlantic Iberian Peninsula	ES-01a	LAM	9	Lamuño	ESP	43.5588388	-6.21887067	119	Juvenile needles
Atlantic Iberian Peninsula	ES-01a	PUE	7	Puerto de Vega	ESP	43.5479494	-6.6313745	81	Juvenile needles

Atlantic Iberian Peninsula	ES-01a	SAC	10	As Neves- San Cipriano de Ribarteme	ESP	42.1183306	-8.36444019	386	Juvenile needles
Atlantic Iberian Peninsula	ES-01a	SEG	19	Sergude (Seed Orchard)	ESP	42.8166667	-8.45	309	Juvenile needles
Atlantic Iberian Peninsula	ES-01a	SIE	8	Sierra de Barcia	ESP	43.5280214	-6.49321942	264	Juvenile needles
Atlantic Iberian Peninsula	PT-04	LEI	21	Leiria	PRT	39.7833333	-8.9575	79	Juvenile needles
**Atlantic Iberian Peninsula**	**PT-04**	**OVA**	**22**	**Ovar**	**PRT**	**40.865**	**-8.56**	**NA**	**Seeds**
Central Spain	ES-02	TBY	22	Tabuyo del Monte	ESP	42.293564	-6.212338	988	Seeds
Central Spain	ES-03	ONA	24	Oña	ESP	42.76264	-3.535043	753	Seeds
Central Spain	ES-03	SOB	24	Sobrón	ESP	42.78947	-3.085837	895	Seeds
Central Spain	ES-04	PFQ	21	Pinofranqueado	ESP	40.365458	-6.38471	724	Seeds
Central Spain	ES-04	SAH	22	El Sahugo	ESP	40.381417	-6.562251	817	Seeds
Central Spain	ES-05	TAL	46	Talayuela	ESP	40.000387	-5.622922	272	Adult needles
Central Spain	ES-06	ARN	17	Arenas de San Pedro	ESP	40.194822	-5.11621336	663	Juvenile needles
Central Spain	ES-06	CEN	9	Cenicientos	ESP	40.2783616	-4.49128075	1079	Juvenile needles
Central Spain	ES-07	VMQ	10	Valdemaqueda	ESP	40.5162521	-4.31113172	947	Juvenile needles
Central Spain	ES-08	BAY	15	Bayubas de Abajo	ESP	41.5229729	-2.87743056	925	Juvenile needles
Central Spain	ES-08	CAR	6	Carbonero el Mayor	ESP	41.1718611	-4.27738333	844	Juvenile needles
Central Spain	ES-08	COC	17	Coca	ESP	41.2547046	-4.49782736	784	Juvenile needles
Central Spain	ES-08	CUE	23	Cuéllar	ESP	41.3746054	-4.48403819	826	Juvenile needles
Central Spain	ES-08	QUI	22	Quintana Redonda	ESP	41.533333	-2.583333	1046	Seeds
Central Spain	ES-09	SAL	10	San Leonardo de Yagüe	ESP	41.8345815	-3.06272011	1070	Juvenile needles
Central Spain	ES-10	COD	22	Codos	ESP	41.282194	-1.417921	1058	Juvenile needles
Central Spain	ES-11	MAZ	21	Mazarete	ESP	40.972458	-2.222067	1171	Juvenile needles
Central Spain	ES-12	BON	8	Boniches	ESP	39.985882	-1.66073269	1090	Seeds

Central Spain	ES-12	SIN	22	Sinarcas	ESP	39.790889	-1.203194	888	Seeds
Central Spain	ES-13	GEA	22	Gea de Albarracín	ESP	40.364782	-1.351394	1367	Seeds
Central Spain	ES-14	OLB	20	Olba	ESP	40.1733093	-0.62296633	989	Seeds
Central Spain	ES-15	AHI	22	Ahín / Eslida	ESP	39.888	-0.332315	733	Juvenile needles
Central Spain	ES-15	MTG	21	Pina de Montalgrao	ESP	40.029711	-0.643612	1148	Adult needles
Central Spain	ES-15	SCD	22	Sierra Calderona	ESP	39.748889	-0.495417	746	Seeds
Central Spain	ES-15	VMA	21	Villamalur	ESP	39.965495	-0.401219	639	Seeds
Central Spain	ES-16	CPA	22	Cortes de Pallás	ESP	39.178116	-0.945833	925	Seeds
Central Spain	ES-A	BEN	22	Benicasim/Benicássim	ESP	40.078744	0.024956	520	Seeds
Central Spain	ES-B	PDL	22	Pradell de la Teixeta	ESP	41.16553	0.865478	560	Seeds
Central Spain	ES-D	QUA	16	Quatretonda	ESP	38.9716451	-0.35884372	420	Adult needles
Corsica	FR-800	ANI	12	Ania	FRA	41.9678121	9.28400271	630	Adult needles
Corsica	FR-800	BAV	16	Bavella	FRA	41.7961125	9.23509536	1020	Adult needles
Corsica	FR-800	BOI	16	Bonifatu	FRA	42.4462715	8.82547096	440	Adult needles
Corsica	FR-800	CAG	11	Cagna	FRA	41.5971554	9.1422139	1040	Adult needles
Corsica	FR-800	GUA	12	Guagno	FRA	42.1730881	8.87852977	500	Adult needles
Corsica	FR-800	PIN	12	Pineto	FRA	42.427234	9.227496	365	Juvenile needles
Corsica	IT-3.2	MON	6	Montignoso	ITA	44.0119027	10.1839092	434	Adult needles
Corsica	IT-4.1	MAG	15	Magra/Montemarcello	ITA	44.0512058	9.970569	100	Adult needles
Corsica	IT-4.2	MUR	7	Murlo	ITA	43.1391764	11.3489856	408	Adult needles
Corsica	IT-4.2	TOC	12	Tocchi	ITA	43.1316902	11.2434269	441	Adult needles
**Fuencaliente**	**ES-E**	**FCN**	**29**	**Fuencaliente**	**ESP**	**38.416503**	**-4.254136**	913	**Adult needles**
Fuencaliente	ES-E	FUEbis	25	Fuencaliente	ESP	38.416503	-4.254136	913	Adult needles
Morocco	MA-01	ADE	21	Adeldal	MAR	35.1385819	-5.08082751	830	Seeds
Morocco	MA-02	KUD	14	Koudiat Erramla	MAR	35.466667	-5.383333	480	Seeds
Morocco	MA-03	MAD	22	Madisouka	MAR	35.1820333	-5.2267	1302	Seeds
Morocco	MA-05	SID	22	Sidi-Meskour	MAR	31.506175	-6.994533	1975	Seeds

Morocco	MA-06	TAJ	22	Tamjout	MAR	33.83333	-3.983333	1500	Seeds
Morocco	MA-07	TAM	14	Tamrabta	MAR	33.6	-5.01666667	1729	Juvenile needles
North East	ES-C	GIR	21	La Bisbal	ESP	41.898572	3.032414	243	Seeds
North East	FR-700	MAU	15	Maures (stand 1)	FRA	43.2332073	6.36536648	361	Adult needles
North East	IT-3.1	ANO	11	Arenzano	ITA	44.41752	8.671061	403	Adult needles
North East	IT-3.1	ROS	10	Rossiglione	ITA	44.5509722	8.64537032	955	Adult needles
North East	IT-3.1	SEB	24	Seborga	ITA	43.82	7.71	544	Adult needles
Point Cires	MA-04	PCI	22	Point Cires	MAR	35.9055533	-5.46339418	90	Seeds
Southeastern Spain	ES-17	CAZ	21	Cazorla	ESP	37.917772	-2.927132	1059	Seeds
Southeastern Spain	ES-17	RIO	24	Riopar	ESP	38.4848	-2.4253	1039	Seeds
Southeastern Spain	ES-19	COM	3	Competa	ESP	36.835601	-3.854090	816	Seeds
Southeastern Spain	ES-19	PEZ	22	La Peza	ESP	37.2738889	-3.36944444	1414	Seeds
Southeastern Spain	ES-20	EST	21	Estepona	ESP	36.516154	-5.120801	458	Adult needles
Southeastern Spain	ES-20	JUB	21	Jubrique	ESP	36.52341	-5.184885	958	Seeds
Southeastern Spain	ES-F	ORI	23	Oria	ESP	37.531165	-2.35113792	1221	Juvenile needles
Southeastern Spain	ES-G	GAU	22	Gaucín	ESP	36.532054	-5.301336	630	Seeds
**Tunisia-Pantelleria**	**AL-01**	**ALG**	**21**	**Algeria**	**ALG**	**36.848**	**4.899**	**154**	**Adult needles**
Tunisia-Pantelleria	IT-7.2	PAN	17	Pantelleria	ITA	36.7917233	11.9999694	733	Adult needles

Tunisia-Pantelleria	TU-01	ABA	22	Aïn Babouch	TUN	36.8189521	8.6787318	201	Seeds
**Tunisia-Pantelleria**	**TU-02**	**TAB**	**22**	**Tabarka**	**TUN**	**36.957**	**8.732**	**222**	**Adult needles**

Supplementary Table S2. Self-assignment tallies using RUBIAS to assign individuals to *Pinus pinaster* populations nested within regions of provenance (RP). The row corresponds to the true source population and the column corresponds to the assigned population. The cells are colored according to the corresponding gene pool. n = number of sampled individuals in population, Hs = expected heterozygosity, pop_specific_fst = population specific F_{ST}.

Note: Table S2 is presented in a separate spreadsheet file (*Supplementary Table S2.xlsx*)

Supplementary Table S3. Average posterior probability for the true source region of provenance (post_mean_pi_mean) and for the true source gene pool (genepool_post_mean_pi) estimated with RUBIAS software for 200-seed *Pinus pinaster* simulated samples originating from a single region of provenance, based on 100 independent replicates per region of provenance, using the default RUBIAS parameter values. n_per_RP = number of individuals in the region of provenance; #RP = number of regions of provenance in the gene pool; n_per_genepool = number of individuals in the gene pool.

RP	n_per_RP	genepool	post_mean_pi_mean	genepool_post_mean_pi	#RP	n_per_genepool
FR303	204	AtlanticFrance	0.986	0.986	2	250
FR700a	46	AtlanticFrance	0.974	0.995	2	250
ES01a	85	AtlanticIberian	0.995	0.995	2	128
PT04	43	AtlanticIberian	0.972	0.995	2	128
ES02	22	CentralSpain	0.995	0.997	18	571
ES03	48	CentralSpain	0.995	0.997	18	571
ES04	43	CentralSpain	0.878	0.973	18	571
ES05	46	CentralSpain	0.974	0.997	18	571
ES06	26	CentralSpain	0.701	0.997	18	571
ES07	10	CentralSpain	0.299	0.997	18	571
ES08	83	CentralSpain	0.984	0.985	18	571
ES09	10	CentralSpain	0.000	0.997	18	571
ES10	22	CentralSpain	0.767	0.997	18	571
ES11	21	CentralSpain	0.946	0.997	18	571
ES12	30	CentralSpain	0.657	0.997	18	571
ES13	22	CentralSpain	0.814	0.997	18	571
ES14	20	CentralSpain	0.792	0.997	18	571
ES15	86	CentralSpain	0.912	0.914	18	571
ES16	22	CentralSpain	0.905	0.997	18	571
ESA	22	CentralSpain	0.950	0.997	18	571
ESB	22	CentralSpain	0.907	0.997	18	571
ESD	16	CentralSpain	0.995	0.997	18	571
FR800	79	Corsica	0.995	0.995	4	119
IT32	6	Corsica	0.000	0.995	4	119
IT41	15	Corsica	0.929	0.995	4	119
IT42	19	Corsica	0.995	0.995	4	119
ESE	54	Fuencaliente	0.995	0.995	1	54
MA01	21	Morocco	0.667	0.996	6	115
MA02	14	Morocco	0.995	0.996	6	115
MA03	22	Morocco	0.773	0.996	6	115
MA05	22	Morocco	0.995	0.996	6	115
MA06	22	Morocco	0.995	0.996	6	115
MA07	14	Morocco	0.995	0.996	6	115
ESC	21	NorthEast	0.951	0.995	3	81

FR700	15	NorthEast	0.869	0.995	3	81
IT31	45	NorthEast	0.927	0.927	3	81
MA04	22	PointCires	0.995	0.995	1	22
ES17	45	SoutheasternSpain	0.995	0.996	5	157
ES19	25	SoutheasternSpain	0.958	0.996	5	157
ES20	42	SoutheasternSpain	0.995	0.996	5	157
ESF	23	SoutheasternSpain	0.995	0.996	5	157
ESG	22	SoutheasternSpain	0.766	0.996	5	157
AL01	21	TunisiaPantelleria	0.618	0.995	4	82
IT72	17	TunisiaPantelleria	0.995	0.995	4	82
TU01	22	TunisiaPantelleria	0.995	0.995	4	82
TU02	22	TunisiaPantelleria	0.814	0.995	4	82
		Average	0.861	0.992		

Supplementary Table S4. A) Self-assignment accuracy using RUBIAS for *Pinus pinaster* baseline gene pools consisting of 1,579 samples genotyped with 10,185 SNP markers. N_same = number of individuals with best assignment in the same gene pool, tot_indv = total number of individuals in the gene pool.

Gene pool	n_same	tot_indv	assign_rate
Atlantic France	248	250	0.992
Atlantic Iberian	153	153	1
Central Spain	566	576	0.983
Corsica	120	120	1
Fuencaliente	54	54	1
Morocco	117	117	1
North East	78	81	0.963
Point Cires	22	22	1
Southeastern Spain	157	157	1
Tunisia-Pantelleria	86	86	1
Average			0.994

B) Test samples that were assigned to an incorrect gene pool. N=number of incorrectly assigned individuals.

Gene pool	Inferred Gene pool	n
Atlantic France	Atlantic Iberian	1
Atlantic France	Southeastern Spain	1
Central Spain	Atlantic Iberian	1
Central Spain	Southeastern Spain	9
North East	Corsica	3

Supplementary Table S5. Expected accuracy of individual genetic assignment to *Pinus pinaster* gene pools, regions of provenance and populations using assignPOP software and a baseline consisting of 1,579 genotypes with 10,185 SNP markers. Based on Monte Carlo cross-validation, using 90% of individuals as training set and 10% as test set, with 100 independent replicates. A) Expected accuracy of assignment to gene pools using assignPOP.

Gene pool	assign_rate
North East	0.974
Central Spain	0.997
Atlantic France	0.990
Corsica	1
Fuencaliente	1
Atlantic Iberian	0.999
Morocco	0.992
Point Cires	1
Tunisia-Pantelleria	1
Southeastern Spain	0.989
Average	0.994

B) Expected accuracy of assignments to regions of provenance (RP) using assignPOP.

RP	assign_rate
AL01	0.710
ES01a	0.999
ES02	1
ES03	1
ES04	1
ES05	0.994
ES06	0.763
ES07	1
ES08	0.986
ES09	0.000
ES10	0.845
ES11	1
ES12	0.907
ES13	0.815
ES14	0.775
ES15	0.994
ES16	0.860
ES17	0.962
ES19	0.903
ES20	1
ESA	0.940
ESB	0.885

ESC	0.945
ESD	0.935
ESE	1
ESF	0.965
ESG	0.810
FR303	0.989
FR700	0.940
FR700a	0.958
FR800	1
IT31	0.970
IT32	0.000
IT41	0.880
IT42	0.995
IT72	1
MA01	0.770
MA02	0.950
MA03	0.695
MA04	1
MA05	1
MA06	1
MA07	1
PT04	0.953
TU01	0.935
TU02	0.800
average	0.888

C) Expected accuracy of assignments to populations using assignPOP. The populations are ordered by their corresponding region of provenance (RP).

Population	assign_rate	RP
ALG	0.685	AL01
ALT	0.570	ES01a
ARM	0.360	ES01a
CAD	0.080	ES01a
CAS	0.060	ES01a
LAM	0.570	ES01a
PUE	0.000	ES01a
SAC	0.150	ES01a
SEG	0.995	ES01a
SIE	0.530	ES01a
TBY	1	ES02
ONA	0.935	ES03
SOB	0.955	ES03
PFQ	0.975	ES04
SAH	1	ES04

TAL	1	ES05
ARN	0.865	ES06
CEN	0.410	ES06
VMQ	1	ES07
BAY	0.280	ES08
CAR	0.440	ES08
COC	0.310	ES08
CUE	0.685	ES08
QUI	1	ES08
SAL	0.020	ES09
COD	0.885	ES10
MAZ	1	ES11
BON	0.790	ES12
SIN	0.840	ES12
GEA	1	ES13
OLB	0.940	ES14
AHI	1	ES15
MTG	0.950	ES15
SCD	1	ES15
VMA	0.990	ES15
CPA	1	ES16
CAZ	0.885	ES17
RIO	1	ES17
COM	0.000	ES19
PEZ	0.975	ES19
EST	0.925	ES20
JUB	0.950	ES20
BEN	1	ESA
PDL	0.925	ESB
GIR	0.945	ESC
QUA	1	ESD
FCN	1	ESE
FUEbis	1	ESE
ORI	1	ESF
GAU	0.970	ESG
HOU	0.623	FR303
LAC	0.340	FR303
LIM	0.803	FR303
MIM	0.065	FR303
OLO	0.560	FR303
PET	0.060	FR303
PLE	0.485	FR303
STJ	0.577	FR303
VER	0.480	FR303
MAU	1	FR700

LCO	0.992	FR700a
ANI	0.900	FR800
BAV	1	FR800
BOI	0.905	FR800
CAG	0.950	FR800
GUA	0.660	FR800
PIN	1	FR800
ANO	0.750	IT31
ROS	0.470	IT31
SEB	1	IT31
MON	0.000	IT32
MAG	0.950	IT41
MUR	0.320	IT42
TOC	1	IT42
PAN	1	IT72
ADE	0.705	MA01
KUD	0.980	MA02
MAD	0.715	MA03
PCI	1	MA04
SID	1	MA05
TAJ	1	MA06
TAM	1	MA07
LEI	0.905	PT04
OVA	1	PT04
ABA	0.975	TU01
TAB	0.835	TU02
average	0.754	

Supplementary Table S6. Average posterior probability of the true *Pinus pinaster* population (post_mean_pi_mean) and region of provenance (RP_post_mean_pi) calculated for simulated samples of size 200 from single populations, applying RUBIAS default values. n_per_pop = number of individuals in the population; #pop = number of populations in the región of provenance; n_per_genepool = number of individuals in the región of provenance.

pop	n_per_pop	RP	post_mean_pi_mean	RP_post_mean_pi	#pop	n_per_RP
ALG	21	AL01	0.616	0.616	1	21
ALT	8	ES01a	0.473	0.867	9	85
ARM	8	ES01a	0.376	0.996	9	85
CAD	8	ES01a	0.156	0.867	9	85
CAS	8	ES01a	0.250	0.996	9	85
LAM	9	ES01a	0.662	0.996	9	85
PUE	7	ES01a	0.000	0.996	9	85
SAC	10	ES01a	0.161	0.996	9	85
SEG	19	ES01a	0.995	0.996	9	85
SIE	8	ES01a	0.127	0.996	9	85
TBY	22	ES02	0.995	0.995	1	22
ONA	24	ES03	0.955	0.995	2	48
SOB	24	ES03	0.872	0.995	2	48
PFQ	21	ES04	0.995	0.995	2	43
SAH	22	ES04	0.953	0.953	2	43
TAL	46	ES05	0.995	0.995	1	46
ARN	17	ES06	0.853	0.853	2	26
CEN	9	ES06	0.217	0.436	2	26
VMQ	10	ES07	0.293	0.293	1	10
BAY	15	ES08	0.201	0.995	5	83
CAR	6	ES08	0.663	0.995	5	83
COC	17	ES08	0.117	0.939	5	83
CUE	23	ES08	0.995	0.995	5	83
QUI	22	ES08	0.951	0.995	5	83
SAL	10	ES09	0.000	0.000	1	10
COD	22	ES10	0.812	0.812	1	22
MAZ	21	ES11	0.995	0.995	1	21
BON	8	ES12	0.127	0.127	2	30
SIN	22	ES12	0.905	0.905	2	30
GEA	22	ES13	0.950	0.950	1	22
OLB	20	ES14	0.995	0.995	1	20
AHI	22	ES15	0.995	0.995	4	86
MTG	21	ES15	0.937	0.937	4	86
SCD	22	ES15	0.995	0.995	4	86
VMA	21	ES15	0.895	0.995	4	86
CPA	22	ES16	0.995	0.995	1	22
CAZ	21	ES17	0.900	0.900	2	45
RIO	24	ES17	0.995	0.995	2	45

COM	3	ES19	0.000	0.995	2	25
PEZ	22	ES19	0.957	0.957	2	25
EST	21	ES20	0.995	0.995	2	42
JUB	21	ES20	0.995	0.995	2	42
BEN	22	ESA	0.995	0.995	1	22
PDL	22	ESB	0.906	0.906	1	22
GIR	21	ESC	0.949	0.949	1	21
QUA	16	ESD	0.995	0.995	1	16
FCN	29	ESE	0.995	0.995	2	54
FUEbis	25	ESE	0.995	0.995	2	54
ORI	23	ESF	0.995	0.995	1	23
GAU	22	ESG	0.904	0.904	1	22
HOU	25	FR303	0.592	0.996	9	204
LAC	21	FR303	0.449	0.996	9	204
LIM	25	FR303	0.837	0.996	9	204
MIM	18	FR303	0.055	0.942	9	204
OLO	24	FR303	0.505	0.996	9	204
PET	22	FR303	0.179	0.996	9	204
PLE	19	FR303	0.285	0.942	9	204
STJ	25	FR303	0.646	0.996	9	204
VER	25	FR303	0.517	0.996	9	204
MAU	15	FR700	0.995	0.995	1	15
LCO	46	FR700a	0.995	0.995	1	46
ANI	12	FR800	0.915	0.995	6	79
BAV	16	FR800	0.995	0.995	6	79
BOI	16	FR800	0.995	0.995	6	79
CAG	11	FR800	0.995	0.995	6	79
GUA	12	FR800	0.859	0.995	6	79
PIN	12	FR800	0.995	0.995	6	79
ANO	11	IT31	0.904	0.995	3	45
ROS	10	IT31	0.397	0.778	3	45
SEB	24	IT31	0.995	0.995	3	45
MON	6	IT32	0.000	0.000	1	6
MAG	15	IT41	0.995	0.995	1	15
MUR	7	IT42	0.000	0.702	2	19
TOC	12	IT42	0.995	0.995	2	19
PAN	17	IT72	0.995	0.995	1	17
ADE	21	MA01	0.666	0.666	1	21
KUD	14	MA02	0.995	0.995	1	14
MAD	22	MA03	0.769	0.769	1	22
PCI	22	MA04	0.995	0.995	1	22
SID	22	MA05	0.995	0.995	1	22
TAJ	22	MA06	0.995	0.995	1	22
TAM	14	MA07	0.995	0.995	1	14
LEI	21	PT04	0.995	0.995	2	43

OVA	22	PT04	0.995	0.995	2	43
ABA	22	TU01	0.995	0.995	1	22
TAB	22	TU02	0.813	0.813	1	22
Average	18.360		0.737	0.913	3.698	62.105

Supplementary Table S7. Self-assignment tallies using RUBIAS to assign individuals to *Pinus pinaster* regions of provenance (RP) nested within gene pools. The row corresponds to the true source region of provenance and the column corresponds to the assigned region of provenance. The cells are colored according to the corresponding gene pool. n = number of individuals in region of provenance.

Note: Table S7 is presented in a separate spreadsheet file (*Supplementary Table S7.xlsx*)
